# A portable immunosensor provides sensitive and rapid detection of *Borrelia burgdorferi* antigen in spiked blood

**DOI:** 10.1038/s41598-023-34108-9

**Published:** 2023-05-09

**Authors:** Sangsik Kim, Kamalika Samanta, Brandon T. Nguyen, Samantha Mata-Robles, Luciana Richer, Jeong-Yeol Yoon, Maria Gomes-Solecki

**Affiliations:** 1 Department of Biomedical Engineering, The University of Arizona, Tucson, AZ 85721 USA; 2 Department of Microbiology, Immunology and Biochemistry, University of Tennessee Health Science Center, Memphis, TN 38163 USA; 3 Present Address: Merck & Co., West Point, PA 19486 USA; 4 Immuno Technologies, Inc, Memphis, TN 38103 USA; 5 College of Medicine, The University of Arizona, Tucson, AZ 85724 USA; 6 Present Address: US Biologic, Inc, Memphis, TN 38103 USA

**Keywords:** Biomedical engineering, Analytical biochemistry, Assay systems

## Abstract

There are no assays for detecting *B. burgdorferi* antigen in blood of infected Lyme disease individuals. Here, we provide proof-of-principle evidence that we can quantify *B. burgdorferi* antigen in spiked blood using a portable smartphone-based fluorescence microscope that measures immunoagglutination on a paper microfluidic chip. We targeted *B. burgdorferi* OspA to develop a working prototype and added examples of two antigens (OspC and VlsE) that have diagnostic value for discrimination of Lyme disease stage. Using an extensively validated monoclonal antibody to OspA (LA-2), detection of OspA antigen had a broad linear range up to 100 pg/mL in 1% blood and the limit of detection (LOD) was 100 fg/mL (= 10 pg/mL in undiluted blood), which was 1000 times lower than our target of 10 ng/mL. Analysis of the two other targets was done using polyclonal and monoclonal antibodies. OspC antigen was detected at LOD 100 pg/mL (= 10 ng/mL of undiluted blood) and VlsE antigen was detected at LOD 1–10 pg/mL (= 0.1–1 ng/mL of undiluted blood). The method is accurate and was performed in 20 min from sample to answer. When optimized for detecting several *B. burgdorferi* antigens, this assay may differentiate active from past infections and facilitate diagnosis of Lyme disease in the initial weeks of infection, when antibody presence is typically below the threshold to be detected by serologic methods.

## Introduction

Lyme disease (LD) is caused by spirochetes of the genus *Borrelia* (*B. burgdorferi* sensu lato) which is transmitted by the bite of an infected *Ixodes* spp. tick^1^. LD is the most common vector-borne disease in the United States and Europe^2,3^ and is widespread in the Northern Hemisphere^4^. As of 2010, the CDC (Centers for Disease Control and Prevention, United States) estimate of annual incidence of clinician-diagnosed LD was ~ 329,000^5^. A recent study by the CDC estimated the annual incidence to be increased to ~ 476,000^6^. Such increase can be attributed to the improved awareness of the disease by the population, better and more frequent laboratory testing, and spread of the disease from its endemic areas in the northeastern United States possibly due to conditions favorable to maintenance of infected ticks in non-endemic areas^7–10^. Early symptoms are benign skin manifestations, i.e., erythema migrans (EM), a clinical marker of LD, which is present in about 60–70% of infected people^11,12^. Severe late disseminated manifestations include neuroborreliosis and arthritis^2,4,13^. However, less than 35% of patients infected with *B. burgdorferi* present with the classic bull’s eye rash^11,12^. The other > 30% present with atypical rashes that are often misdiagnosed^14^, thereby putting this group of patients, in addition to the group that does not develop EM (> 30%)^15^, at risk for developing late LD. Therefore, prompt diagnosis and treatment of LD are critical to preventing permanent damage to the nervous and musculoskeletal systems.

The CDC recommends identifying the disease through a standard two-tiered (STT) serologic assay algorithm^16^ or by using a modified two-tiered testing (MTTT) protocol for detection of specific antibody to *B. burgdorferi*^17,18^. However, the overall sensitivity for detection of early *B. burgdorferi* infection within the first weeks post development of EM rash is very low, 14–17%^19,20^. Although these serodiagnostic protocols are highly specific and have high sensitivity (> 85%) after the first 3 weeks post presentation of EM and other symptoms^19^, this type of indirect test does not reliably discriminate between active infection and past exposure, unless paired serum samples can be tested longitudinally to demonstrate a > fourfold decline in IgG^21^. Paired collection of samples is not routinely done for LD^21^.

Direct methods of detection that target *B. burgdorferi* in biological samples that can be used for diagnosis of LD include direct visualization of the bacteria after staining^22^, culture of the spirochete in BSK-H media^23^, nucleic acid amplification tests (NAATs) such as blood PCR and solid tissue or body fluid PCR^17,24,25^, metagenomics such as targeted deep sequencing^21^, and detection of OspA antigen^26^. However, direct visualization of *B. burgdorferi* in blood or other tissues as well as *B. burgdorferi* culture are not routinely done given the low burden of *B. burgdorferi* in vivo, as well as the long culture incubation periods of up to 6 weeks^21^. Transient, low burden of organisms in blood is also the reason that PCR of *B. burgdorferi* nucleic acids is challenging^21,27^, although sometimes useful to help diagnose Lyme arthritis^28^.

One way to address these limitations is to develop a *B. burgdorferi* antigen-detection assay that can perform with a very low limit of detection (LOD) at the earliest phase of infection (1–3 weeks post tick bite and/or EM) using biological samples routinely used such as blood.

Previously, our group demonstrated a novel method of counting immunoagglutinated fluorescent nanoparticles one by one (particulometry) on a paper-based microfluidic chip using a portable smartphone-based fluorescence microscope. It performed with a very low LOD (down to a single virus copy) and high specificity^29–31^. We adapted this method for detection of *B. burgdorferi-*specific antigens in blood. Although OspA has been detected in urine and serum of LD patients^26,32^ and OspC antigen has been detected in cutaneous biopsies of patients with LD^33^, detection of *B. burgdorferi* antigens of conventional diagnostic significance (e.g., OspC and VlsE) have not been demonstrated in blood. To demonstrate proof-of-principle of this new technology and develop a working prototype, we evaluated the performance (low LOD and broad linear range) of OspA antigen–antibody pairs in diluted blood, a standard method modification used for these applications. In addition, we did a preliminary analysis of two antigens that have diagnostic value for discrimination of Lyme disease stage: outer surface protein C (OspC) and Variable lipoprotein surface Exposed (rVlsE).

## Results

### Overview of the assay prototype

**Figure 1:**
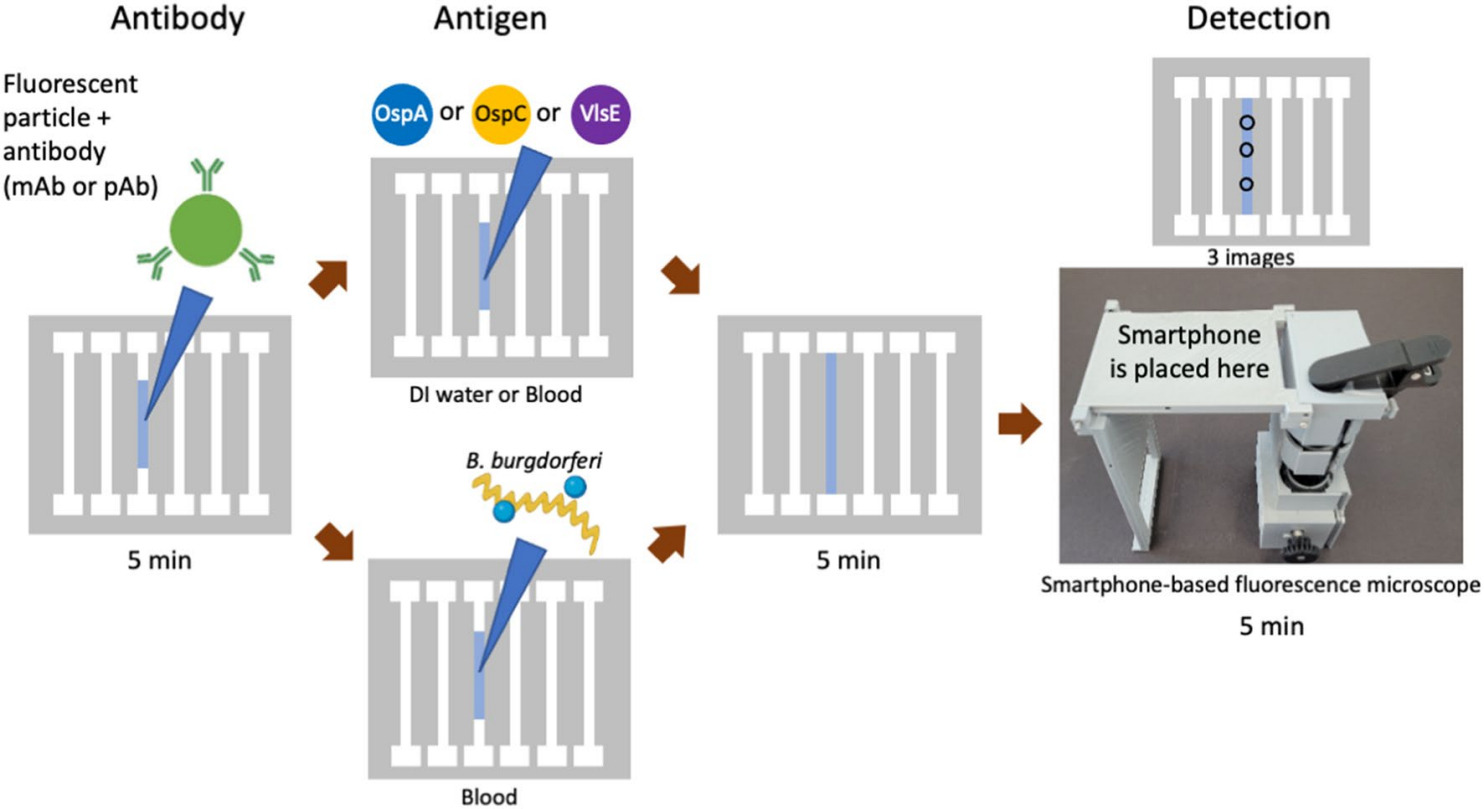
Overall experimental design and assay procedure. *DI* deionized; *OspA* outer surface protein A; *OspC* outer surface protein C; *VlsE* variable lipoprotein surface exposed; *mAb* monoclonal antibody; *pAb* polyclonal antibody.

We used monoclonal antibodies (mAb) to OspA and VlsE, and a polyclonal antibody (pAb) to a cocktail of 8 OspCs (A, B, C, D, H, I, K, and N) to detect *B. burgdorferi* recombinant proteins (rOspA, rVlsE, and rOspC type K) spiked in deionized water and blood diluted to 1%. Inactivated *B. burgdorferi* expressing OspA was also tested in spiked 1% blood. Figure 1 shows the overall experimental design and assay procedure. Briefly, 3 μL of antibody-conjugated fluorescent particles was added to each channel’s center, which filled ~ 60% of the channel. After drying for 5 min, 3 μL of antigen- or inactivated *B. burgdorferi*-spiked deionized water or blood samples was added to the center and allowed to fill the entire channel. After drying for 5 min, we used a custom-made smartphone-based fluorescence microscope to image three different areas of a single channel, and counted the pixel sums of the immunoagglutinated particles. An intensity threshold was used to eliminate background noise, and a size threshold was used to isolate only the immunoagglutinated particles.

### Specificity of antigen–antibody pairs

**Figure 2:**
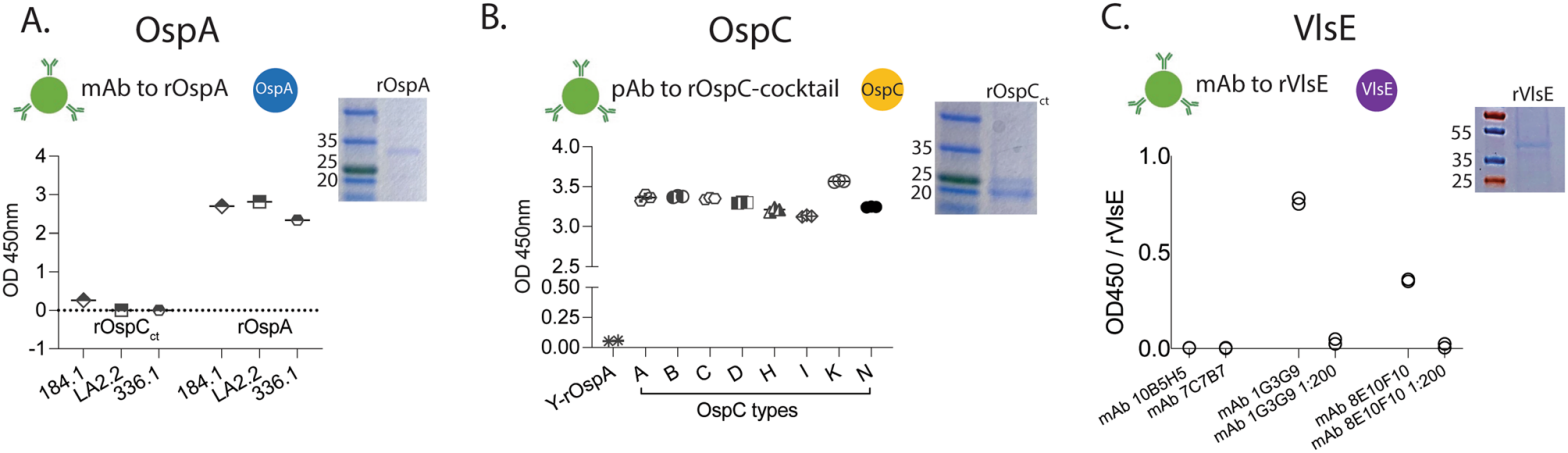
Specificity of antigen–antibody pairs used to develop a new portable immunosensor assay for detection of *B. burgdorferi* antigen in biological fluids. (**A**) Average of four reactions of 3 anti-OspA monoclonal antibodies (184.1, LA2.2, and 336.1) to rOspC_ct_ (control, 23 kDa) and rOspA (32 kDa). (**B**) Reactivity of a polyclonal antibody generated to a cocktail of 8 OspC_ct_ against each recombinant OspC type and to yeast puried recombinant OspA (Y-rOspA control). (**C**) Reactivity of 4 monoclonal antibody hybridoma supernatant genarated to rVlsE (36 kDa; 10B5H5, 7C7B7, 1G3G9, and 8E10F10) against puried recombinant VlsE. *kDa* kilodalton; *mAb* monoclonal antibody. Raw SDS-PAGE images are available as Supplementary Figs. S1 and S2.

Coomassie blue staining of SDS-PAGE gels of the three recombinant proteins confirmed its purity and expected molecular weight: OspA, ~ 32 kDa; OspC, ~ 23 kDa; and VlsE, ~ 36 kDa (Fig. 2). The reactivity of each of the purified recombinant proteins to the respective specific monoclonal and polyclonal antibodies, and controls, was tested by ELISA and is shown in Fig. 2. Monoclonal antibodies to OspA (184.1, LA2.2, and 336.1) reacted with rOspA but not with rOspC (Fig. 2A); polyclonal antibody cocktail to 8 OspC antigens reacted to all *E. coli* purified recombinant OspCs used to produce the anti-sera, including OspC_K_, but not to recombinant OspA purified from yeast (control) (Fig. 2B). Of the 4 anti-VlsE hybridoma supernatants screened against rVlsE, two specific monoclonal antibodies (1G3G9 and 8E10F10) tested positive and were selected for further purification and analysis (Fig. 2C).

### Detection of recombinant OspA in deionized (DI) water and spiked blood

**Figure 3:**
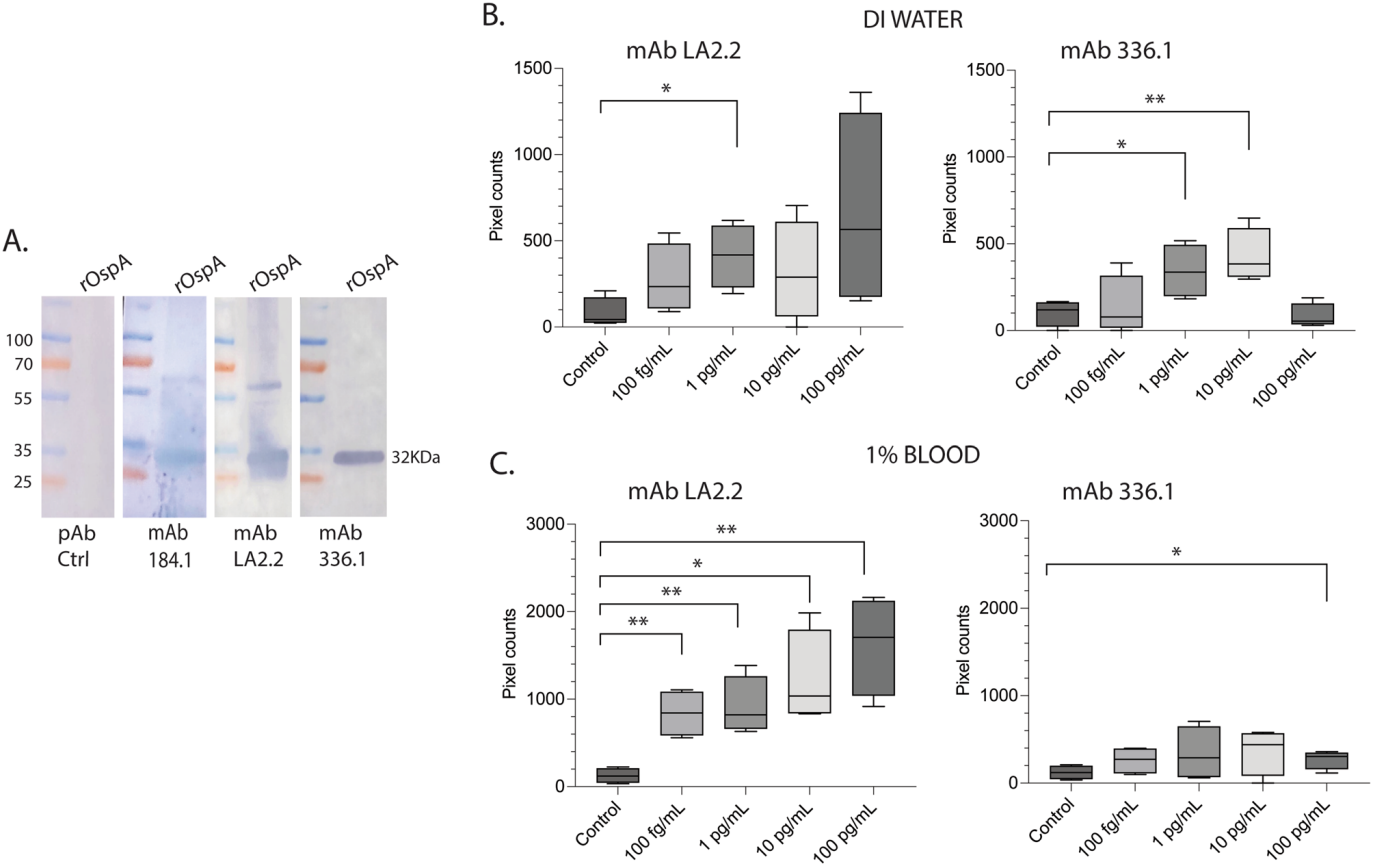
Detection of *B. burgdorferi* recombinant OspA (rOspA) in deionized (DI) water and human blood (1%) by a new portable immunosensor. (**A**) specificity of rOspA was further analyzed by western blot against pAb control and mAb 184.1, LA2.2, and 336.1. (**B** and **C**) Box and whiskers plot representing the average and standard error from three independent experiments of rOspA detection by LA2.2 and 336.1 mAbs in DI water (**B**) and 1% blood (**C**) using a different paper microfluidic channel each time. Significant differences between the control and test channel by Student’s t-test, *p < 0.05, **p < 0.01. *pAb* polyclonal antibody; *mAb* monoclonal antibody; *kDa* kilodalton. Raw western blot images are available as Supplementary Fig. S3.

The recombinant OspA protein (rOspA) binding to specific monoclonal antibodies was further confirmed by western blot (Fig. 3A). Purified rOspA was subsequently solubilized in Tween 20-treated deionized (DI) water and spiked in 1% human blood in concentrations ranging from 100 fg/mL to 100 pg/mL to be tested in the portable immunosensor assay using anti-OspA monoclonal antibodies LA2.2 and 336.1 conjugated to fluorescent particles. In DI water (Fig. 3B), mAb LA2.2 detection showed an overall increasing trend up to 100 pg/mL, with significant differences from control at 1 pg/mL (*p* = 0.0143). In addition, mAb 336.1 showed linear detection of rOspA up to 10 pg/mL, with significant differences from control at 1 pg/mL (*p* = 0.0218) and at 10 pg/mL (*p* = 0.0082). The LOD of the assay in DI water was identical for both mAbs, at 1 pg/mL. In 1% blood (Fig. 3C), a linear increase was observed for detection of rOspA with mAb LA2.2 with significant differences between the control and the four concentrations of rOspA, 100 fg/mL, *p* = 0.0045; 1 pg/mL, *p* = 0.0072; 10 pg/mL, *p* = 0.0125; and 100 pg/mL, *p* = 0.0062. mAb 336.1 detected rOspA in 1% blood in the 100 pg/mL range with *p* = 0.0341. We also tested lower OspA concentrations using the LA2.2 antibody. However, none of the lower concentrations were significantly different from the control, setting the LOD at 100 fg/mL for rOspA detection with mAb LA2.2. This LOD in 1% blood corresponds to 10 pg/mL in 100% blood.

### Detection of *B. burgdorferi* bacteria in spiked blood

**Figure 4:**
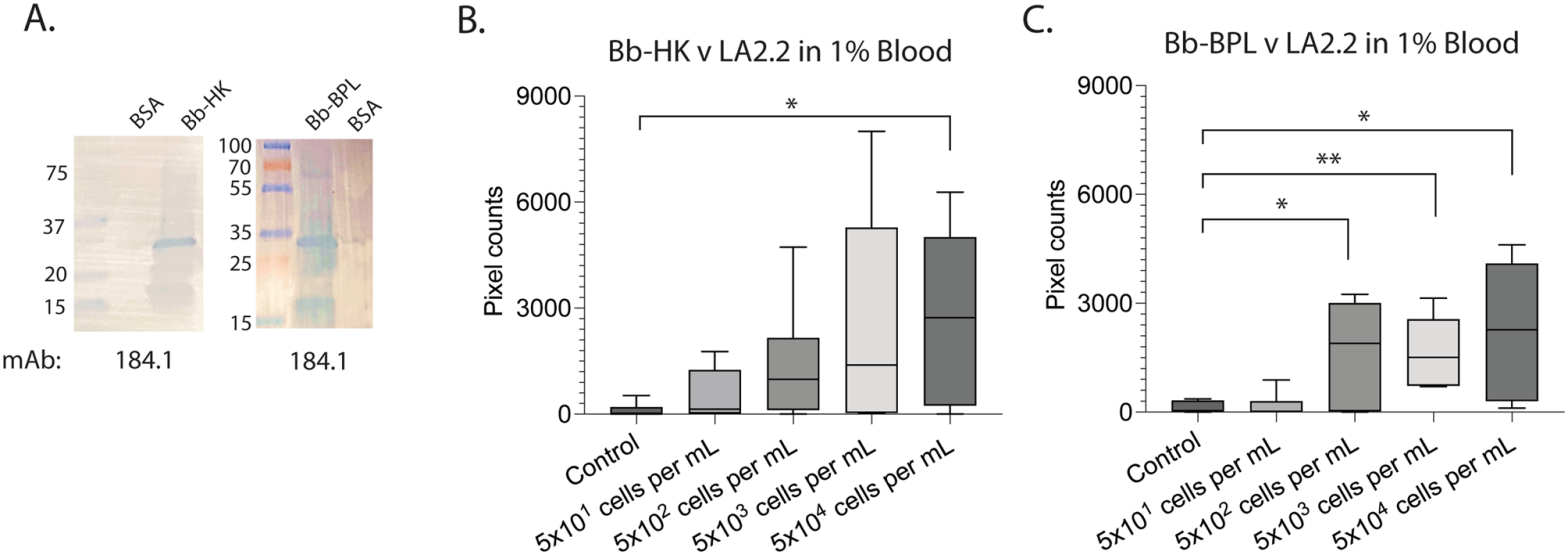
Detection of inactivated *B. burgdorferi* in human blood (1%) by a new portable immunosensor. (**A**) Western blot confirmation of OspA expression in *B. burgdorferi* cultures inactivated by heat-killing (HK) and by treatment with BPL (beta-propiolactone) using anti-OspA mAb 184.1. (**B**) Box and whiskers plot representing the average and standard error from three independent experiments of HK- and BPL-treated *B. burgdorferi* cultures in 1% human blood using a different paper microfluidic channel each time. Significant differences between the control and test channel by Student’s t-test, *p < 0.05, **p < 0.01. *Bb Borrelia burgdorferi*; *mAb* monoclonal antibody; *kDa* kilodalton. Raw western blot images are available as Supplementary Figs. S4 and S5.

We tested blood spiked with inactivated cells of *B. burgdorferi* grown in culture conditions permissive to expression of OspA, using the monoclonal antibody that performed better in previous analyses (mAb LA2.2). The bacteria cultures were inactivated by heat killing (HK) and by beta-Propiolactone (BPL) treatment and OspA expression was confirmed by western blot using mAb 184.1, an antibody distinct from the mAb used in the immunosensor assay (Fig. 4A). The inactivated bacteria were spiked into 1% human blood. An increasing trend was observed for both HK- and BPL-treated bacterial samples, with comparable extents of pixel counts. However, the error bars of the heat-killed (HK) reactions were substantial, rendering differences between control and test samples mostly non-significant, except for 5 × 10^4^ cells/mL with *p* = 0.0260 (Fig. 4B). In contrast, reactions between LA2.2 and BPL-inactivated *B. burgdorferi* (Fig. 4C) were significantly different from the control: 5 × 10^2^ cells/mL, *p* = 0.0237; 5 × 10^3^ cells/mL, *p* = 0.0055; and 5 × 10^4^ cells/mL, *p* = 0.0232. The LOD of this assay was 500 BPL-inactivated *B. burgdorferi* cells per mL.

### Detection of recombinant OspC and VlsE in spiked blood

**Figure 5:**
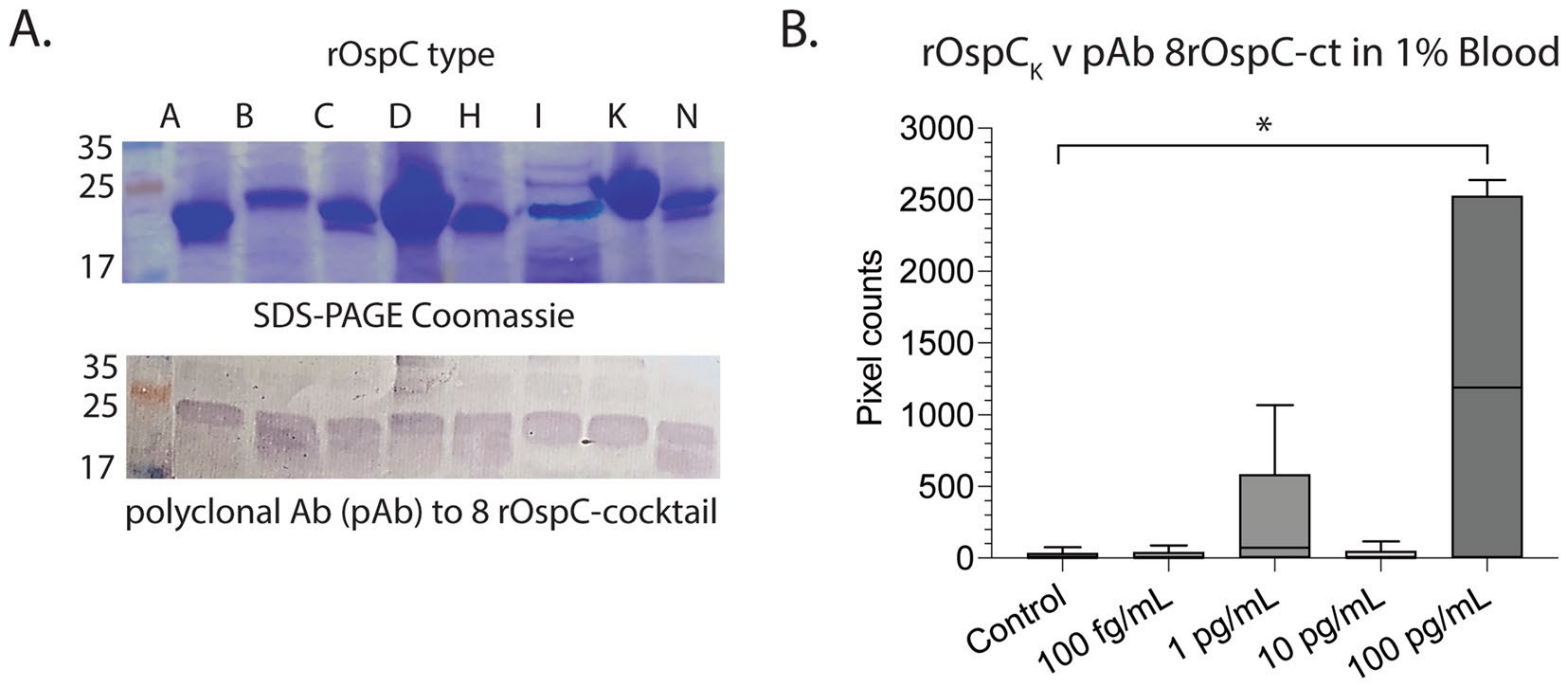
Detection of recombinant *B. burgdorferi* OspCK in human blood (1%) by a new portable immunosensor. (**A**) Western blot analysis of the rOspC-cocktail polyclonal antibody against the 8 purified OspC proteins (pAb 8rOspC-ct); (**B**) Box and whiskers plot representing the average and standard error from three independent experiments of rOspCK detection by pAb 8rOspC-ct using a different paper microfluidic channel each time. Differences between the control and test channel by Student’s t-test, *p* < 0.05. Raw SDS-PAGE images are available as Supplementary Figs. S6 and S7.

**Figure 6:**
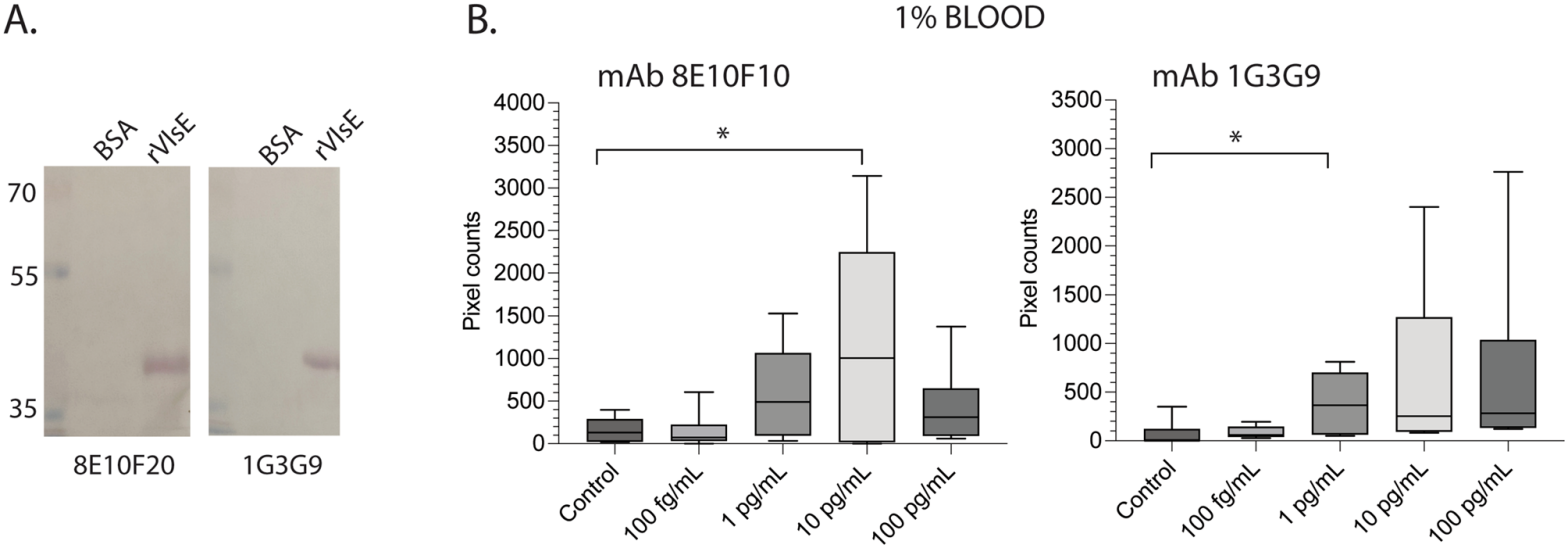
Detection of *B. burgdorferi* recombinant VlsE (rVlsE) in human blood (1%) by a new portable immunosensor. (**A**) rVlsE was first analyzed by western blot against mAbs 1G3G9 and 8E10F10. (**B**) Box and whiskers plot representing the average and standard error from three independent experiments of rVlsE detection by the same mAbs in 1% blood using a different paper microfluidic channel each time. Significant differences between the control and test channel by Student’s t test, *p < 0.05. Raw western blot images are available as Supplementary Fig. S8.

We did a preliminary analysis of two additional targets (rOspC and rVlsE) to be included in the core of a future immunosensor (Figs. 5 and 6, respectively). For recombinant OspC detection, we used a polyclonal antibody (pAb) generated against a cocktail of 8 OspC types (A, B, C, D, H, I, K, and N). We confirmed the binding of the polyclonal antibody to all 8 purified OspC recombinant proteins electrotransferred to a PVDF (polyvinylidene fluoride) membrane by western blot (Fig. 5A) before conjugating the antibody with fluorescent particles. Recombinant OspC type K protein (rOspC_K_) was spiked in 1% human blood in concentrations ranging from 100 fg/mL to 100 pg/mL to be tested in the portable immunosensor assay using the polyclonal antibody cocktail (pAb 8rOspC-ct), (Fig. 5B). The anti-OspC pAb detected the highest concentration of rOspC_K_ tested (100 pg/mL) with *p* = 0.0381. To detect rVlsE two monoclonal antibodies conjugated to the fluorescent particles were used (mAb 8E10F10 and 1G3G9) (Fig. 6). A linearly increasing trend is observed with anti-VlsE mAb 8E10F10 from 100 fg/mL to 10 pg/mL, with the lowest concentration of 10 pg/mL statistically different from the control (*p* = 0.0472), i.e., the LOD (equivalent to 1 ng/mL in undiluted blood). A broader linear trend can be observed with anti-VlsE mAb 1G3G9 from 100 fg/mL to 100 pg/mL, with a lower LOD of 1 pg/mL at *p* = 0.0342 (equivalent to 100 pg/mL in undiluted blood).

## Discussion

Direct detection of antigen in biological samples defines an assay that helps diagnosis of active infections, in contrast to antibody detection which does not discriminate between present (active) and past infections. Unlike nucleic acid amplification tests, methods that target the pathogen proteins directly in the blood of LD patients detect viable spirochetes and have the revolutionary advantage of producing a decisive result with diagnostic value within the first weeks post-infection. Here, we present the proof-of-principle for an assay capable of detecting *B. burgdorferi* antigen and whole cells in biological fluids using a smartphone microscope and a paper microfluidic chip.

We chose *B. burgdorferi* outer surface proteins OspA, OspC, and VlsE as antigen targets for the following reasons. OspA is the ideal target for proof-of-principle analysis of new technology because we have monoclonal antibodies (184.1, LA-2, and 336.1) validated using other platforms^34–39^ and we can easily grow *B. burgdorferi* expressing OspA in culture, to evaluate the presence of whole spirochetes. We chose mAbs LA2 and 336.1 to set up the assay in the immunosensor because they bind to the protective C-terminus of OspA^37,40^, thus these mAbs define well-exposed epitopes in OspA. Furthermore, OspA has been used to develop diagnostic assays for detection of *B. burgdorferi* antigen in clinical samples^32,41^ and some of those assays have been approved by the FDA. OspC is necessary for *B. burgdorferi* invasion of the vertebrate host early in infection, which indicates this antigen is a good target to identify early LD infections^42,43^. Furthermore, OspC antigen has been detected in infected mouse tissues and plasma^44^ and in cutaneous biopsies from LD patients. VlsE is expressed by *B. burgdorferi* throughout the course of infection^45,46^, which indicates this protein is an excellent candidate to identify early, disseminated, and late Lyme disease stages. In addition, *B. burgdorferi* can be grown in special culture conditions to express OspC and VlsE^47^, which allows for further refinement of the technology for detecting live *B.burgdorferi* if applicable.

In the first set of experiments, we tested two monoclonal antibodies targeting *B. burgdorferi* recombinant OspA in a simple fluid of DI water and a complex biological fluid of blood, diluted to 1%. For this class of assay, whole blood is traditionally diluted to 10% and/or 1%, because whole blood viscosity prevents flow in microfluidics^48,49^. In our experiment, 1% blood sample had better linearity than DI water. This pattern has often been seen in previous studies as well^48,49^. In a study that detected ROR1 + cancer cells in buffy coat blood samples, the linearity of aggregation did not increase with cell concentration. This is because the presence and properties of blood components affect the pattern of antigen–antibody aggregation and the flow rate (distance).

In DI water, the LOD for detection of rOspA using both mAb (LA2.2 and 336.1) was 1 pg/mL (= 3 fg per sample, considering the sample volume of 3 μL) (Fig. 3B). Interestingly, the LOD improved to 100 fg/mL (= 0.3 fg per sample) for detection of rOspA by the LA-2.2 antibody in 1% blood (Fig. 3C), but not by mAb 336.1. The components in the blood might have contributed to stabilizing the particles and promoting antibody-antigen binding. LA-2 is a very well-characterized monoclonal antibody that binds to a protective B cell epitope in the larger C-terminus of OspA (aa208-253)^36,37^. Thus, it is an epitope well exposed and available for recognition by the antibody. In contrast, mAb 336.1 binds to the adjacent left side of the LA-2 binding site (~ aa 260-273) near the very C-terminus helix of OspA^50^. Thus, this epitope may have not been as available for avid binding by the antibody. In other studies, quantification of OspA in serum was achieved by multiple reaction monitoring mass spectometry using samples from 3 LD patients^26^. The LOD was 4.0 fmol of OspA per mg of serum protein, which is equivalent to 112 ng per g serum protein. Our LOD is significantly lower by several orders of magnitude. A downside of these other methods is the requirement for expensive laboratory equipment and highly trained personnel. Our assay has the advantage of performance speed—it can be done within 20 min. The device is also portable and low-cost, requiring a smartphone, a smartphone microscope attachment, an LED, and an acrylic film.

To further interrogate the useability of this technology, we tested blood spiked with inactivated cells of *B. burgdorferi* grown in culture conditions permissive to the expression of OspA (Fig. 4), using the monoclonal antibody that showed a broader linear range in previous analyses (mAb LA2.2, Fig. 3C). *B. burgdorferi* cultures were inactivated in two ways, by heat kill (HK) inactivation and by beta-propiolactone (BPL) inactivation (Fig. 4). The LOD of the BPL-inactivated *B. burgdorferi* was 5 × 10^2^ cells/mL (= 1.5 cells per sample, considering the sample volume of 3 μL), i.e., at a single copy level, whereas the LOD of the HK-inactivated *B. burgdorferi* was much higher at 5 × 10^4^ cells/mL. The difference between the inactivation methods is important for developing a diagnostic assay, given that heat inactivation kills the bacteria causing denaturation of the proteins, whereas BPL inactivation kills the bacteria while preserving the integrity of the membrane. After infecting a vertebrate host, *B. burgdorferi* will be attacked by immune cells, and we would expect a combination of *B. burgdorferi* structures to be available in blood or plasma for detection. Targeting other proteins such as OspC and VlsE is necessary to increase the assay sensitivity and could lead to development of antigen assays able to discriminate early from late Lyme disease.

Antigen detection assays that target OspA in biological samples have not been tested enough to be accepted by the medical community. Those assays that target OspA in urine are commercially available but are not routinely done given that OspA expression by *B. burgdorferi* is downregulated during infection of the host^21^. Thus, targeting other *B. burgdorferi* biomarkers in blood will contribute to the design of robust antigen detection assays for diagnosis of early to late stage Lyme disease. To do this, we started by targeting OspC and VlsE. The OspC antigen is important for development of LD diagnostics because it is expressed when *B. burgdorferi* infects a vertebrate host. This protein works very well to detect antibodies to *B. burgdorferi* in human serum and has been used to develop diagnostic assays for early LD^45,51–54^. We were able to detect rOspC in 1% diluted blood at a LOD of 100 pg/mL (~ 10 ng/mL of undiluted blood) using a polyclonal antibody that was generated to 8 types of *B. burgdorferi* OspC (Fig. 5). The data shows that OspC antigen–antibody complexes can be detected in blood. This LOD is equivalent to our target of 10 ng/mL^44^. The fact that we reached our target using a polyclonal antibody suggests that we should be able to vastly improve this LOD when we use a good monoclonal antibody to OspC. VlsE is expressed upon *B. burgdorferi* infection and is a robust biomarker for all stages of LD diagnostics^45,53^. *B. burgdorferi* VlsE was also detected in 1% blood using two monoclonal antibodies (8E10F10 and 1G3G9) at LOD 1–10 pg/mL (equivalent to 0.1–1 ng/mL of undiluted blood). To date, detection of VlsE in blood has not been shown but it is reasonable to define a threshold of 10 ng/mL based on OspC. Additional work needs to be done on OspC and VlsE to produce monoclonal antibodies that will match or surpass the LOD achieved for OspA detection, which was 100 fg/mL. Overall, our data provides evidence that the assay can be optimized to target multiple proteins of *B. burgdorferi* in blood.

## Conclusion

In summary, our assay detected *B. burgdorferi* antigens in spiked blood samples using a low-cost, portable smartphone-based fluorescence microscope and a paper microfluidic chip. The total time to complete the assay was 20 min. Among the various antibodies tested to OspA, OspC, and VlsE, anti-OspA mAb LA2.2 showed the best assay performance. The LOD was 100 fg/mL OspA in 1% blood, equivalent to 10 pg/mL in undiluted whole blood, which is substantially lower than many other portable immunosensors^31,55^ and is 1000 times below our target of 10 ng/mL. The LOD of bacteria cells was 5 × 10^2^ cells/mL (= 1.5 cells per sample), i.e., a single copy level. Further work needs to be done to select better mAbs for detection of OspC, VlsE and other antigens (i.e., p66 and DbpA) at the same LOD reached for OspA to develop highly sensitive assays that discriminate between early and late stages of LD. Furthermore, the method can easily be adapted for antibody detection, similar to the existing serologic assays. Both antigen and antibody assays could be conducted in a single paper microfluidic chip to provide a more comprehensive diagnostic result.

## Methods

### *Borrelia burgdorferi* proteins and antibodies

Recombinant OspA, OspC, and VlsE of *B. burgdorferi* were overexpressed in pET9a/c (OspA/OspC) and pET28a (VlsE) vectors and the proteins were purified. Briefly, IPTG-induced *Escherichia coli* transformed with the gene of interest were grown at 37 °C, 200 rpm, in TBY (tryptone broth yeast) supplemented with 50 μg/mL kanamycin to OD_600_ of 1. The cells were harvested by centrifugation at 9000× *g* for 20 min at 4 °C, washed with PBS, weighed and the pellets were resuspended with BugBuster Master Mix (MilliporeSigma, Burlington, MA, USA) supplemented with protease inhibitor cocktail (Complete, Roche Diagnostics GmBH, Germany) at a volume of 5 mL/gr. The pellets were disrupted by frequent vortexing and incubating in a shaker for 30 min at room temperature, and subjected to a final centrifugation step. Cell lysates containing the recombinant protein were purified by anion-exchange chromatography using Q-sepharose Fast Flow resin (Cytiva Marlborough, MA, USA) in TBST buffer (pH 9.2–9.5) at 4 °C. The protein was eluted off the column with 0.5 M NaCl in TBST. The eluate was passed through the Pierce Detergent Removal Spin Columns (Thermo Fisher Scientific, Waltham, MA, USA) and the protein concentration was determined by the Detergent Compatibility assay method. Protein size and purity was checked by Coomassie staining of SDS-PAGE gels (8–16%, Mini-Protean, Bio-Rad Laboratories, Hercules, CA, USA) and electrotransferred to PVDF membranes to be subjected to Western blot with specific monoclonal antibodies.

The full sequence of *B. burgdorferi* VlsE was sent to Genscript Biotech Corp. for contract production of anti-VlsE monoclonal antibodies. Previously characterized monoclonal antibodies to OspA 184.1, LA2.2, and 336.1^37,38^, and 4 new monoclonal antibodies to VlsE (10B5H5, 7C7B7, 8E10F10, and 1G3G9) were purified from hybridoma supernatants using MAbTrap Kit (Cytiva, Marlborough, MA, USA). In addition, polyclonal OspC (antibody cocktail) antiserum was produced by vaccinating C3H-HeJ mice every other week for 6 weeks using 50 μg of purified OspC cocktail that included OspC types A, B, C, D, H, I, K, and N.

Simulated samples were created by spiking the purified recombinant proteins to the commercially available whole human blood diluted to 1% (from pooled donors (catalog number 010-ABSH-PMG, Winchester, VA, USA). Antigen concentrations were set up from 100 fg/mL to 10 ag/mL by serial dilutions.

No human participants were involved in the study. All methods were carried out in accordance with relevant guidelines and regulations. All experimental protocols were conducted in a biosafety level 2 laboratory, approved by the Research Laboratory & Safety Services (RLSS) at the University of Arizona.

### Culture of *B. burgdorferi* expressing OspA and inactivation methods

A culture of *B. burgdorferi* obtained by heart culture of C3H-HeN mice challenged with ticks flagged in the field sites in NY, USA between 2005 and 2008 was maintained by passage in the Gomes-Solecki laboratory and confirmed to contain 10 OspC types of *B. burgdorferi* by NGS sequencing^56^. This multistrain culture (MS05-08) was grown to mid-log phase (~ 5 × 10^7^ cells) at 34 °C in complete BSK-H medium at pH 7.5 supplemented with 6% normal rabbit serum and antibiotic (1:100 from laboratory stock) for ~ 1 week.

Beta-propiolactone (BPL) inactivation: BPL was added to 10^8^ cells culture of *B. burgdorferi* and was kept in a shaker at 34 °C for 24 h; the process was repeated 24 h later after which 10 µL of the culture was checked under a dark field microscope to confirm absence of motile of *B. burgdorferi;* 50 µL was inoculated into BSK-H media and cultured for 1 week; samples were collected on days 1, 3 and 7 to confirm absence of bacterial growth by FlaB qPCR. The culture was centrifuged at 12,000 g at 10 °C for 15 min and washed with PBS 3 times; after the last centrifugation the culture was suspended in 500 µL of PBS and stored at −80 °C until use.

Heat inactivation: 10^8^ cells of *B. burgdorferi* culture was incubated at 56 °C for 1 h; 10 µL of culture was checked under a dark field microscope to confirm lack of mobility; 50uL was inoculated into BSK-H media and cultured for 1 week; samples were collected on days 1, 3, 7 to confirm absence of bacterial growth by FlaB qPCR; the culture was centrifuged at 12,000 g, 10 °C, 15 min and washed in PBS 3 times; after the last wash the pellets was resuspended in 500 µL of PBS and stored at − 80 °C until use.

### Paper microfluidic chips

The paper microfluidic chip was designed in SolidWorks 2020 software (Dassault Systèmes, Vélizy-Villacoublay, France) and wax printed on nitrocellulose paper using ColorCube 8550 (Xerox, Norwalk, CT, USA) as described previously^57^. Each chip contains four flow channels that measure 21 mm long and 2.4 mm wide, with larger square-shaped loading pads at each end. The nitrocellulose paper (FF80HP Plus; GE Healthcare, MA, USA) had a capillary flow rate of 60–100 s over 40 mm and a thickness of 200 μm.

### Antibody-conjugated fluorescent particles

Each type of antibody was covalently conjugated to 0.5-µm diameter yellow-green fluorescent, carboxylated polystyrene particles (Magsphere, Inc., Pasadena, CA, USA). The peak excitation wavelength was 488 nm. Only one type of antibody was conjugated to each batch of particles. The covalent conjugation protocol can be found at https://doi.org/10.17504/protocols.io.bhsvj6e6.

### Assay procedure

Each antigen concentration was assayed three times, each time using a different channel. A single chip could test two different antigen concentrations since it had six channels. 3 μL of antibody-conjugated fluorescent particles was added to each channel’s center and spread via capillary action. Since the volume was insufficient to fill the entire length of a channel, it filled ~ 60% of the channel. It was left to dry for 5 min. After drying, 3 μL of antigen sample (in DI water or 1% whole blood) was added to the center of each channel and spread via capillary action until it filled the entire length of the channel. During this period, antigens reacted with the previously loaded antibody-conjugated particles, resulting in immunoagglutination. It was again left to dry for 5 min. About 15 min from addition of the antibody-conjugated particles, the chip was loaded into the chip holder and imaged following the protocol outlined below.

### Smartphone-based fluorescence microscopy of paper microfluidic chips

Fluorescence microscopic images of each paper microfluidic channel were captured using a smartphone-based fluorescence microscope, described in^30^. It utilized a commercial microscope attachment to a smartphone (MicroFlip 100–250 × High Power Pocket Microscope; Carson Optical, NY, USA). A 460 nm LED (WP7113QBC/G; Digi-Key Electronics, MN, USA) was used as a light source for the excitation of fluorescent particles. The excitation wavelength is slightly shorter than the peak excitation of the particles (488 nm) to avoid the overlap with the emission signal while providing sufficient excitation to the particles, as confirmed by the fluorescence images. A 9-V battery powered this LED. An acrylic film (Color Filter Booklet; Edmund Optics, AZ, USA) with a cut-on wavelength of 500 nm was used as the low-cost emission filter placed between the microscope attachment and smartphone camera. As discussed below, a smartphone camera (Samsung Galaxy S20 FE 5G; Samsung Electronics America, Inc., NJ, USA) was used to image each paper channel and isolate only the aggregated particles. All components (a microscope attachment, a LED, a 9-V battery, an acrylic filter, and a smartphone) were mounted on a foldable stand and a stage designed in SolidWorks and 3D-printed using Creality Ender-3 (Shenzhen Creality 3D Technology Co. Ltd.; Shenzhen, Guangdong, China) with PETG filament (Overture; Wilmington, DE, USA). Smartphone images were taken using ProCam 4 app with 1/60 shutter speed, 4000 white balance, and 400 ISO.

### Image processing using MATLAB

The MATLAB (The MathWorks, Inc.; Natick, MA, USA) script was used to process the images, publicly available in the Supplementary Data 2 of^30^. Since each microscopic image’s field-of-view (FOV) was too small to capture the overall particle aggregation behavior, three different locations were imaged for a single channel. The custom code written in MATLAB analyzed these three images from each channel. Initially, the image was separated into red, green, and blue (RGB) channel images. We used green channel images to represent the green emission of particles. The intensities < 50 were considered noise and removed. Next, we eliminated the particles whose pixel sizes < 21, i.e., the particles ≥ 21 were considered aggregated, confirmed with the benchtop fluorescence microscopic images^30^. The pixel areas of all aggregated particles were summed for a given image.

### Statistics

The Student’s t test (equal variances) was used to compare each concentration with the control (zero-antigen or zero-bacteria spiked). While error bars seemed varied, the true variances (when adjusted to normal distribution, i.e., standard errors divided by means) are roughly equivalent, i.e., less than 4–5 times of differences.

## Supplementary Information

Supplementary Information.

## Data Availability

All data generated or analyzed during this study are included in this manuscript. Raw SDS-PAGE and Western blot images are available in the Supplementary Figs. S1-S9. Raw images of smartphone-based fluorescent microscopy are available upon request by emailing J.-Y.Y. (jyyoon@arizona.edu).

## References

[1] Eldin C (2019). Review of European and American guidelines for the diagnosis of Lyme borreliosis. Med. Mal. Infect..

[2] Shapiro ED, Gerber MA (2000). Lyme disease. Clin. Infect. Dis..

[3] Centers for Disease Control and Prevention (CDC). Lyme disease–United States, 2001–2002. *MMWR Morb. Mortal. Wkly. Rep.* **53**, 365–369 (2004). 15129194

[4] Stanek G, Wormser GP, Gray J, Strle F (2012). Lyme borreliosis. Lancet.

[5] Nelson CA (2015). Incidence of clinician-diagnosed Lyme disease, United States, 2005–2010. Emerg. Infect. Dis..

[6] Schwartz AM, Kugeler KJ, Nelson CA, Marx GE, Hinckley AF (2021). Use of commercial claims data for evaluating trends in Lyme disease diagnoses, United States, 2010–2018. Emerg. Infect. Dis..

[7] Schwartz AM, Hinckley AF, Mead PS, Hook SA, Kugeler KJ (2017). Surveillance for Lyme disease - United States, 2008–2015. MMWR Morb. Mortal. Wkly. Rep..

[8] Foster E (2022). Inter-annual variation in prevalence of *Borrelia burgdorferi* sensu stricto and *Anaplasma phagocytophilum* in host-seeking *Ixodes scapularis* (Acari: Ixodidae) at long-term surveillance sites in the upper midwestern United States: Implications for public health practice. Ticks Tick-borne Dis..

[9] Kugeler KJ, Farley GM, Forrester JD, Mead PS (2015). Geographic distribution and expansion of human Lyme disease United States. Emerg. Infect. Dis..

[10] Ogden NH, Mechai S, Margos G (2013). Changing geographic ranges of ticks and tick-borne pathogens: Drivers, mechanisms and consequences for pathogen diversity. Front. Cell. Infect. Microbiol..

[11] Steere AC, Schoen RT, Taylor E (1987). The clinical evolution of Lyme arthritis. Ann. Intern. Med..

[12] Marques AR (2010). Lyme disease: A review. Curr. Allergy Asthma Rep..

[13] Hengge UR (2003). Lyme borreliosis. Lancet Infect. Dis..

[14] Schutzer SE (2013). Atypical erythema migrans in patients with PCR-positive Lyme disease. Emerg. Infect. Dis..

[15] Steere AC (1989). Lyme disease. N. Engl. J. Med..

[16] Centers for Disease Control and Prevention (CDC). Recommendations for test performance and interpretation from the Second National Conference on Serologic Diagnosis of Lyme Disease. *MMWR Morb. Mortal. Wkly. Rep.* **44**, 590–591 (1995). 7623762

[17] Moore A, Nelson C, Molins C, Mead P, Schriefer M (2016). Current guidelines, common clinical pitfalls, and future directions for laboratory diagnosis of Lyme disease, United States. Emerg. Infect. Dis..

[18] Brandt KS, Horiuchi K, Biggerstaff BJ, Gilmore RD (2019). Evaluation of patient IgM and IgG reactivity against multiple antigens for improvement of serodiagnostic testing for early Lyme disease. Front. Public Health..

[19] Wormser GP (2008). Impact of clinical variables on *Borrelia burgdorferi*-specific antibody seropositivity in acute-phase sera from patients in North America with culture-confirmed early Lyme disease. Clin. Vaccine Immunol..

[20] Steere AC, McHugh G, Damle N, Sikand VK (2008). Prospective study of serologic tests for Lyme disease. Clin. Infect. Dis..

[21] Branda JA, Steere AC (2021). Laboratory diagnosis of Lyme borreliosis. Clin. Microbiol..

[22] Aberer E, Duray PH (1991). Morphology of *Borrelia burgdorferi*: Structural patterns of cultured borreliae in relation to staining methods. J. Clin. Microbiol..

[23] Pollack RJ, Tedford SR, Spielman A (1993). Standardization of medium for culturing Lyme disease spirochetes. J. Clin. Microbiol..

[24] Babady NE, Sloan LM, Vetter EA, Patel R, Binnicker MJ (2008). Percent positive rate of Lyme real-time polymerase chain reaction in blood, cerebrospinal fluid, synovial fluid, and tissue. Diagn. Microbiol. Infect. Dis..

[25] Schutzer SE (2019). Direct diagnostic tests for Lyme disease. Clin. Infect. Dis..

[26] Cheung CSF (2015). Quantification of *Borrelia burgdorferi* membrane proteins in human serum: A new concept for detection of bacterial infection. Anal. Chem..

[27] Eshoo MW (2012). Direct molecular detection and genotyping of *Borrelia burgdorferi* from whole blood of patients with early Lyme disease. PLoS ONE.

[28] Li X (2011). Burden and viability of *Borrelia burgdorferi* in skin and joints of patients with erythema migrans or lyme arthritis. Arthritis Rheum..

[29] Chung S (2019). Smartphone-based paper microfluidic particulometry of norovirus from environmental water samples at the single copy level. ACS Omega.

[30] Chung S (2021). Norovirus detection in water samples at the level of single virus copies per microliter using a smartphone-based fluorescence microscope. Nat. Protoc..

[31] Breshears L (2022). Sensitive, smartphone-based SARS-CoV-2 detection from clinical saline gargle samples. PNAS Nexus.

[32] Magni R (2015). Application of Nanotrap technology for high sensitivity measurement of urinary outer surface protein A carboxyl-terminus domain in early stage Lyme borreliosis. J. Transl. Med..

[33] Schnell G (2015). Discovery and targeted proteomics on cutaneous biopsies infected by *Borrelia* to investigate Lyme disease. Mol. Cell Proteom..

[34] Del Rio B (2008). Oral immunization with recombinant Lactobacillus plantarum induces a protective immune response in mice with Lyme disease. Clin. Vaccine Immunol..

[35] Del Rio B (2010). Immune response to Lactobacillus plantarum expressing *Borrelia burgdorferi* OspA is modulated by the lipid modification of the antigen. PLoS ONE.

[36] Johnson B (1995). Incomplete protection of hamsters vaccinated with unlipidated OspA from *Borrelia burgdorferi* infection is associated with low levels of antibody to an epitope defined by mAb LA-2. Vaccine.

[37] Ding W (2000). Structural identification of a key protective B-cell epitope in Lyme disease antigen OspA. J. Mol. Biol..

[38] McGrath B (1995). Identification of an immunologically important hypervariable domain of major outer surface protein A of *Borrelia burgdorferi*. Infect. Immun..

[39] Gomes-Solecki M (2006). Oral vaccine that breaks the transmission cycle of the Lyme disease spirochete can be delivered via bait. Vaccine.

[40] Koide S (2005). Structure-based design of a second-generation Lyme disease vaccine based on a C-terminal fragment of *Borrelia burgdorferi* OspA. J. Mol. Biol..

[41] Tabb JS, Rapoport E, Han I, Lombardi J, Green O (2022). An antigen-targeting assay for Lyme disease: Combining aptamers and SERS to detect the Osp protein. Nanomed. Nanotechnol. Biol. Med..

[42] Fung B (1994). Humoral immune response to outer surface protein C of *Borrelia burgdorferi* in Lyme disease: Role of the immunoglobulin M response in the serodiagnosis of early infection. Infect. Immun..

[43] Tily K (2006). *Borrelia burgdorferi* OspC protein required exclusively in a crucial early stage of mammalian infection. Infect. Immun..

[44] Dolange V, Simon S, Morel N (2021). Detection of *Borrelia burgdorferi* antigens in tissues and plasma during early infection in a mouse model. Sci. Rep..

[45] Arumugam S (2019). A multiplexed serologic test for diagnosis of Lyme disease for point-of-care use. J. Clin. Microbiol..

[46] Jacek E (2016). Epitope-specific evolution of human B cell responses to *Borrelia burgdorferi* VlsE protein from early to late stages of Lyme disease. J. Immunol..

[47] Zhang Y (2020). YebC regulates variable surface antigen VlsE expression and is required for host immune evasion in *Borrelia burgdorferi*. PLoS Pathog..

[48] Ulep T (2020). Smartphone based on-chip fluorescence imaging and capillary flow velocity measurement for detecting ROR1+ cancer cells from buffy coat blood samples on dual-layer paper microfluidic chip. Biosens. Bioelectron..

[49] Zenhausern R (2022). Natural killer cell detection, quantification, and subpopulation identification on paper microfluidic cell chromatography using smartphone-based machine learning classification. Biosens. Bioelectron..

[50] Huang X (1998). NMR identification of epitopes of Lyme disease antigen OspA to monoclonal antibodies. J. Mol. Biol..

[51] Akarapipad P (2022). Smartphone-based sensitive detection of SARS-CoV-2 from saline gargle samples via flow profile analysis on a paper microfluidic chip. Biosens. Bioelectron..

[52] Arnaboldi PM (2013). Outer surface protein C peptide derived from *Borrelia burgdorferi* sensu stricto as a target for serodiagnosis of early Lyme disease. Clin. Vaccine Immunol..

[53] Nayak S (2016). Microfluidics-based point-of-care test for serodiagnosis of Lyme disease. Sci. Rep..

[54] Radtke FA (2021). Serologic response to *Borrelia* antigens varies with clinical phenotype in children and young adults with Lyme disease. Clin. Microbiol..

[55] Lahey LJ (2015). Development of a multiantigen panel for improved detection of *Borrelia burgdorferi* infection in early Lyme disease. J. Clin. Microbiol..

[56] Melo R (2016). Oral immunization with OspC does not prevent tick-borne *Borrelia burgdorferi* infection. PLoS ONE.

[57] Kim S, Romero-Lozano A, Hwang D, Yoon J-Y (2021). A guanidinium-rich polymer as a new universal bioreceptor for multiplex detection of bacteria from environmental samples. J. Hazard. Mater..

