# Supplementary material for: A portable immunosensor provides sensitive and rapid detection of Borrelia burgdorferi antigen in spiked blood
Source: Sci Rep. 2023 May 9;13:7546. doi: 10.1038/s41598-023-34108-9 (PMC10170079; doi:10.1038/s41598-023-34108-9)
Supplement: Supplementary file 1 — Supplementary Information. [file 41598_2023_34108_MOESM1_ESM.docx]

**SUPPLEMENTARY INFORMATION**

**A portable immunosensor provides sensitive and rapid detection of *Borrelia burgdorferi* antigen in spiked blood**

Sangsik Kim^1,#^, Kamalika Samanta^3,#,a^, Brandon T. Nguyen^2^, Samantha Mata-Robles^1^, Luciana Richer^3b^, Jeong-Yeol Yoon^1,*^ and Maria Gomes-Solecki^3,*^

^1^Department of Biomedical Engineering, The University of Arizona, Tucson, Arizona 85721, United States

^2^College of Medicine, The University of Arizona, Tucson, Arizona 85724, United States

^3^Department of Microbiology, Immunology and Biochemistry, University of Tennessee Health Science Center, Memphis, Tennessee 38163, United States

^a^ Present address: Merck & Co., West Point, PA, United States

^b^ Present address: US Biologic, Inc., Memphis, TN, United States

^#^ Co-first authors

^*^Corresponding authors. E-mails: [jyyoon@arizona.edu](mailto:jyyoon@arizona.edu) (J.-Y.Y.) and [mgomesso@uthsc.edu](mailto:mgomesso@uthsc.edu) (M.G.-S.).


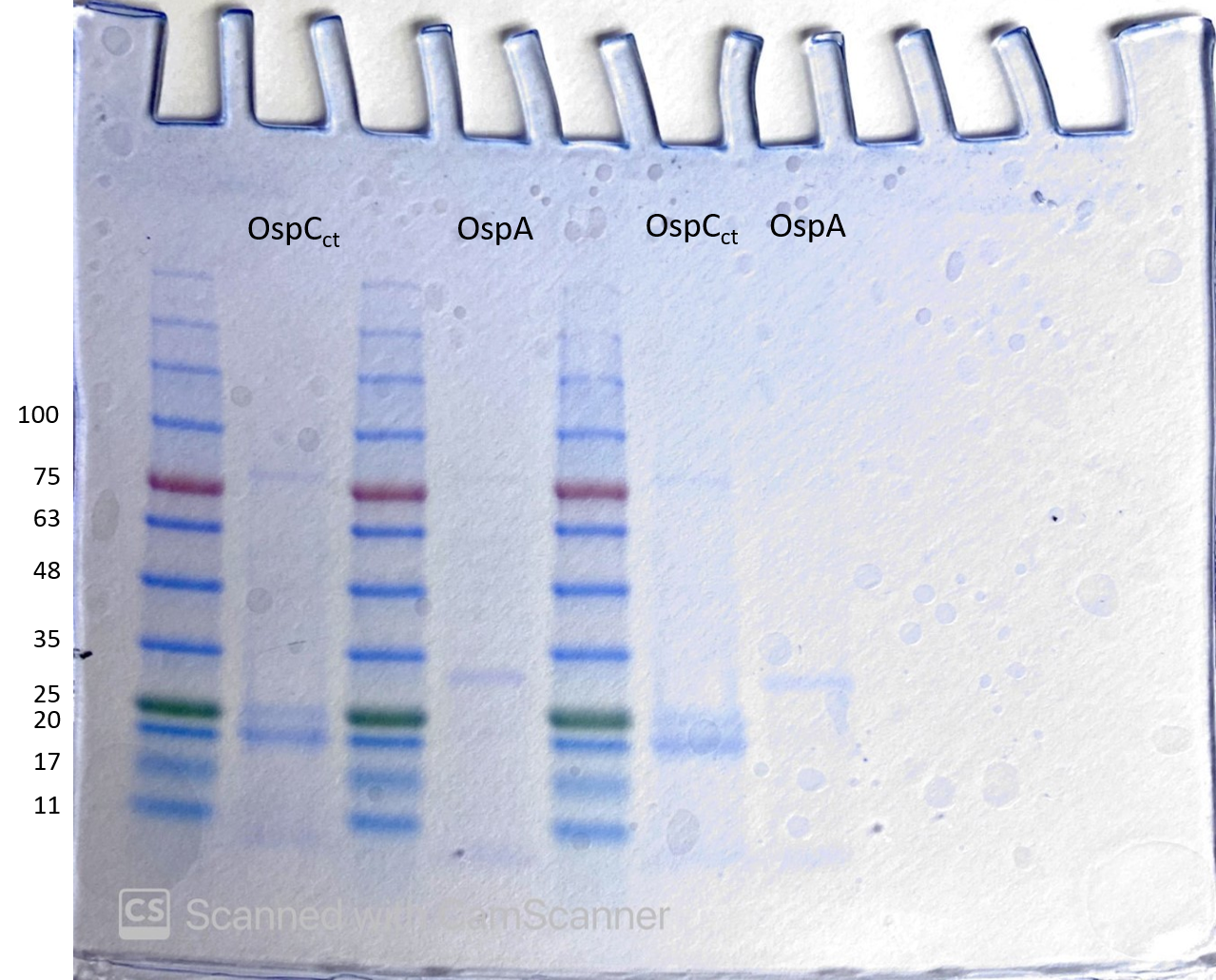


**Supplementary Figure S1.** Raw image for Figures 2A and 2B. Coomassie blue staining of SDS-PAGE of rOspA (~32 kDa) and rOspC_ct_ (~23 kDa).


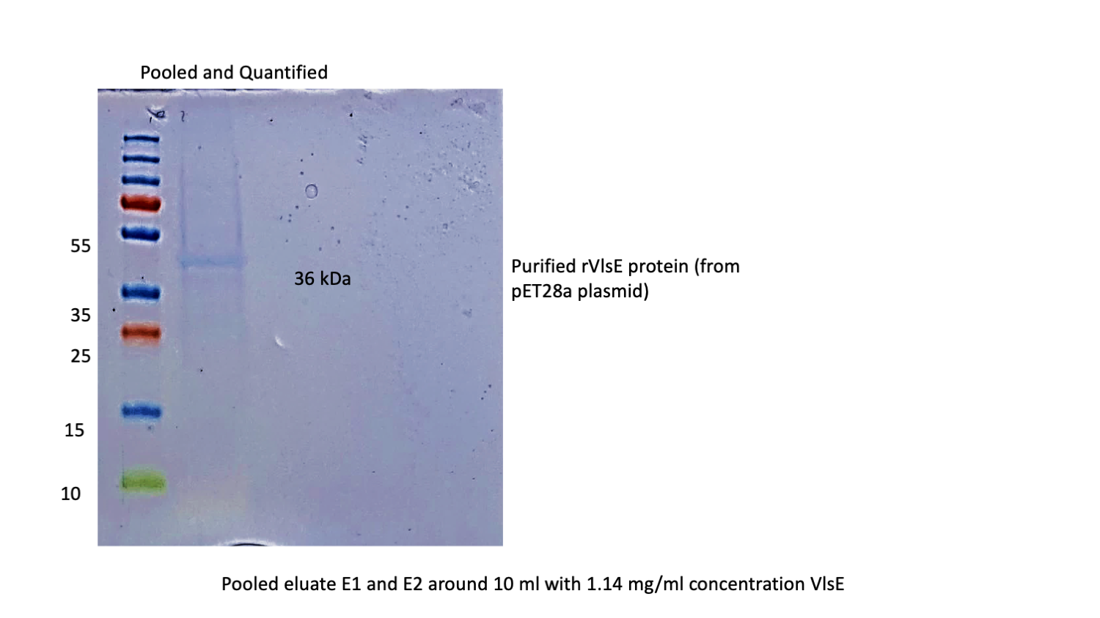


**Supplementary Figure S2.** Raw image for Figure 2C. Coomassie blue staining of SDS-PAGE of purified rVlsE protein from pET28a plasmid (~36 kDa). Pooled eluate E1 and E2 around 10 mL with 1.14 mg/mL concentration VlsE.


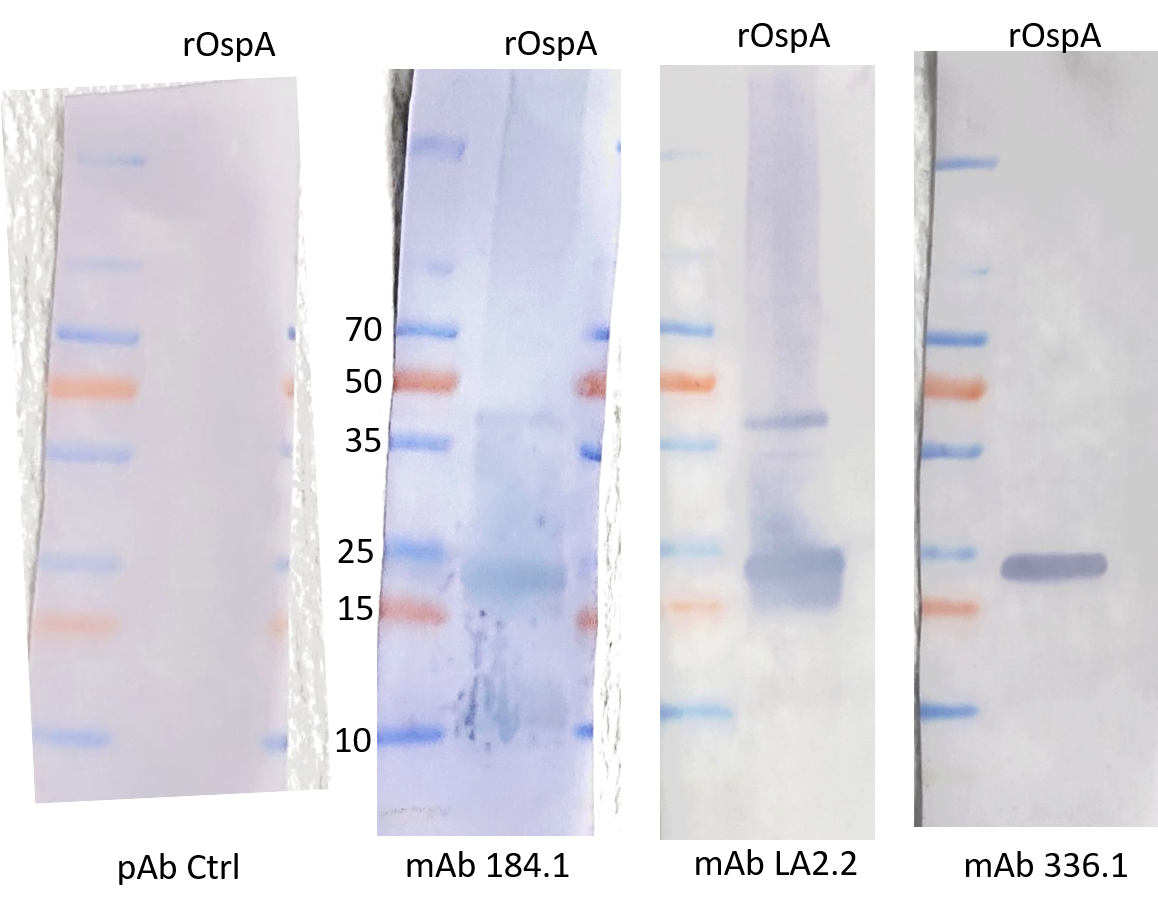


**Supplementary Figure S3.** Raw images for Figure 3A. Western blot of PVDF (polyvinylidene fluoride) electrotransferred rOspA vs. serum control (pAb control) and OspA mAbs.


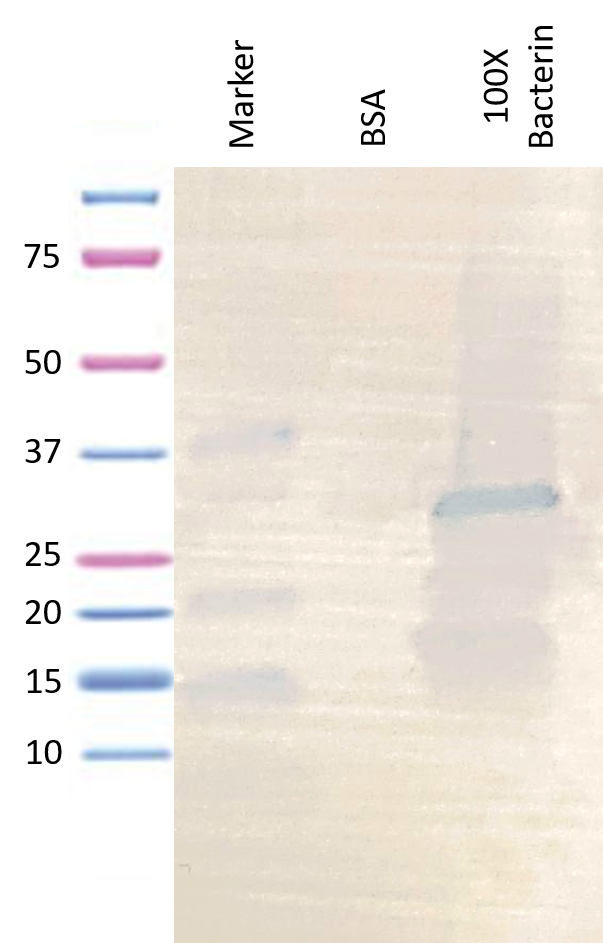


**Supplementary Figure S4.** Raw image for Figure 4A. Western blot of PVDF electrotransferred heat-killed *B. burgdorferi* vs. OspA mAb 184.1.


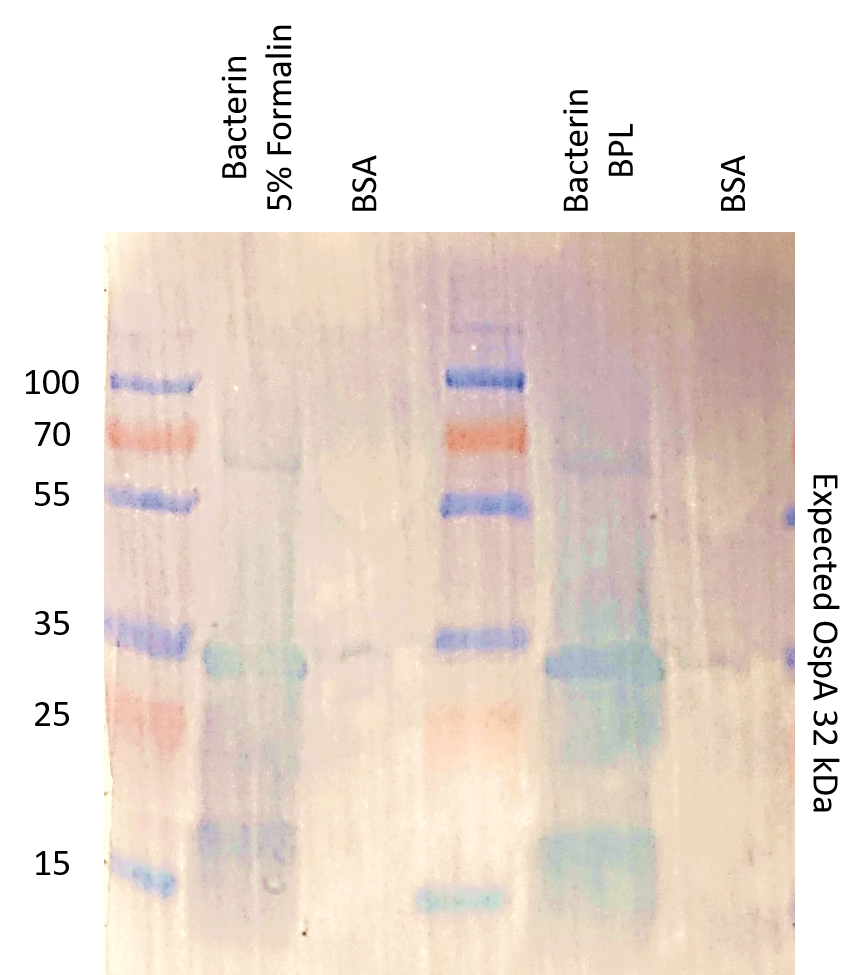


**Supplementary Figure S5.** Raw image for Figure 4A. Western blot of PVDF electrotransferred beta-propiolactone killed *B. burgdorferi* vs. OspA mAb 184.1. 10X bacterin loaded. Primary mAb (OspA 184.1) = 1 : 1000.


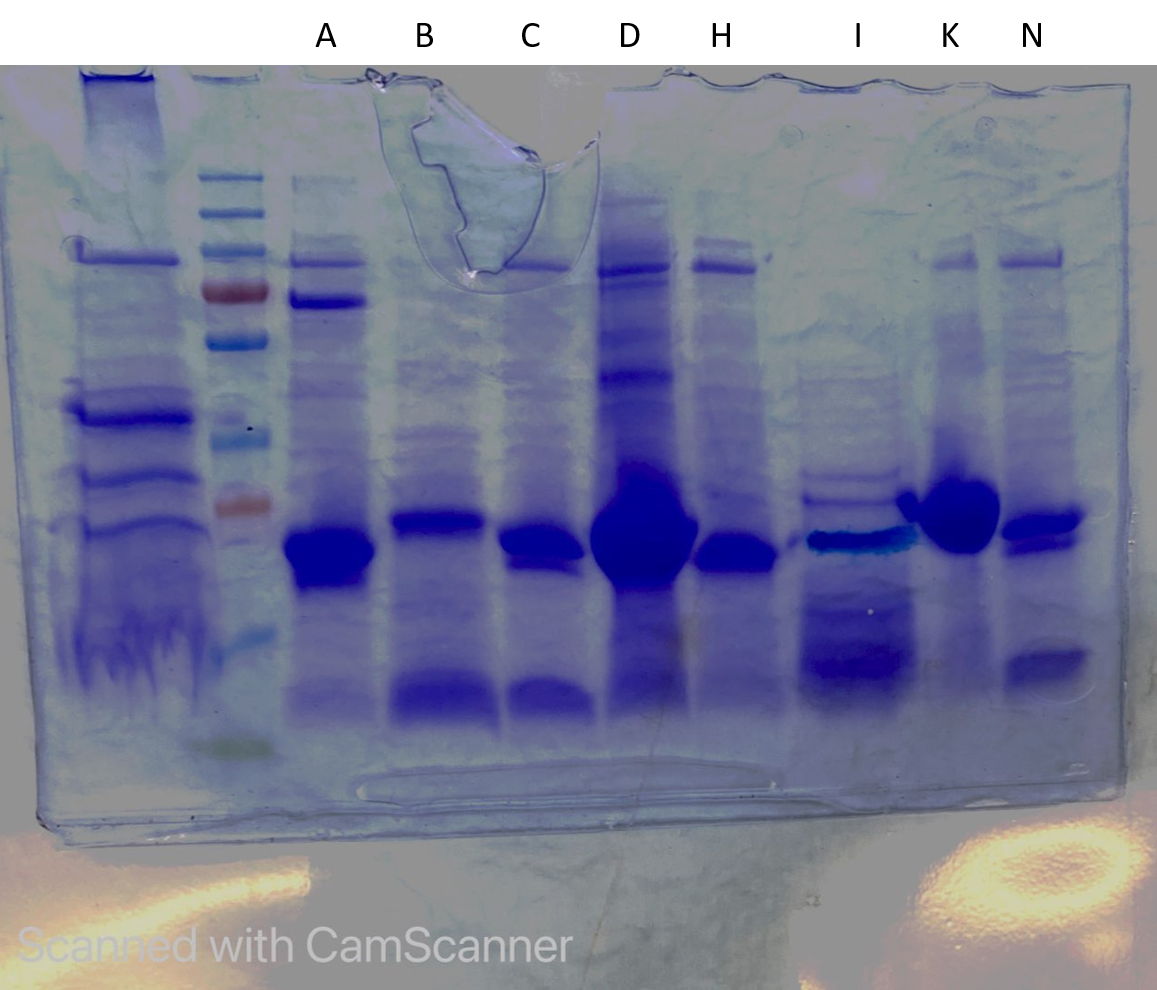


**Supplementary Figure S6.** Raw image for Figure 5A. Coomassie blue staining of SDS-PAGE of 8 OspC’s.


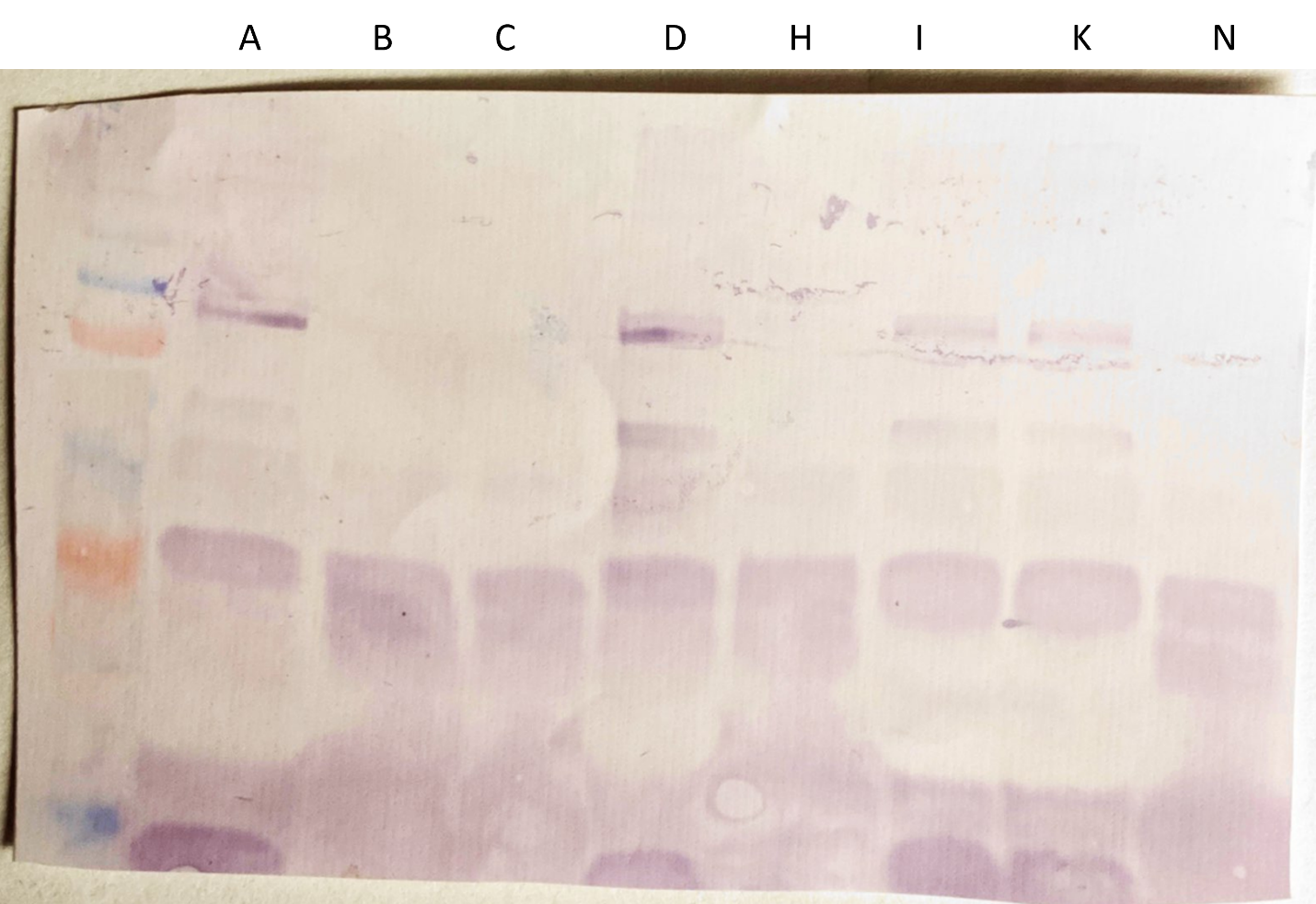


**Supplementary Figure S7.** Raw image for Figure 5A. Western blot of PVDF electrotransferred 8 OspC’s vs. anti-OspC cocktail polyclonal antibody.


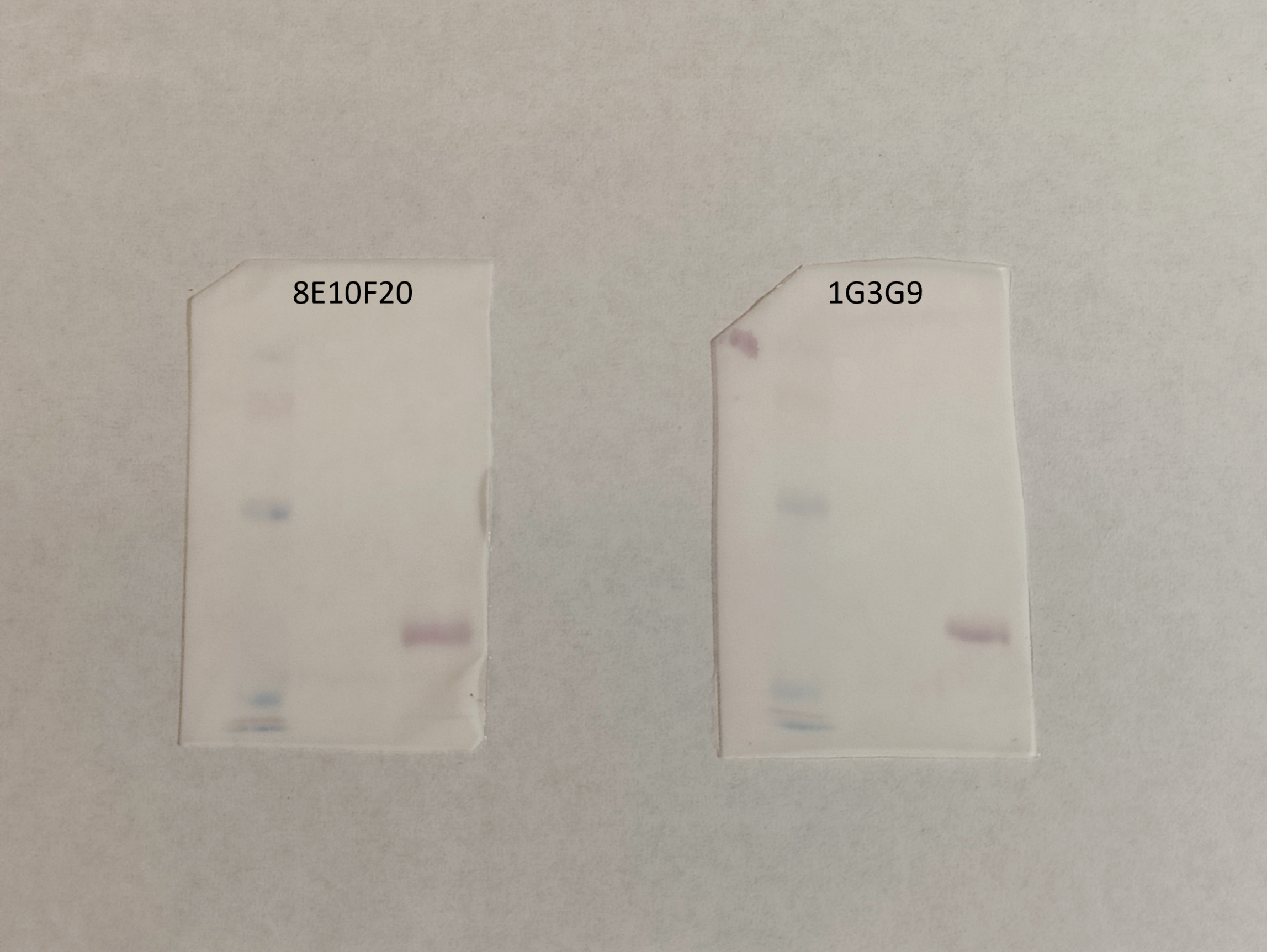

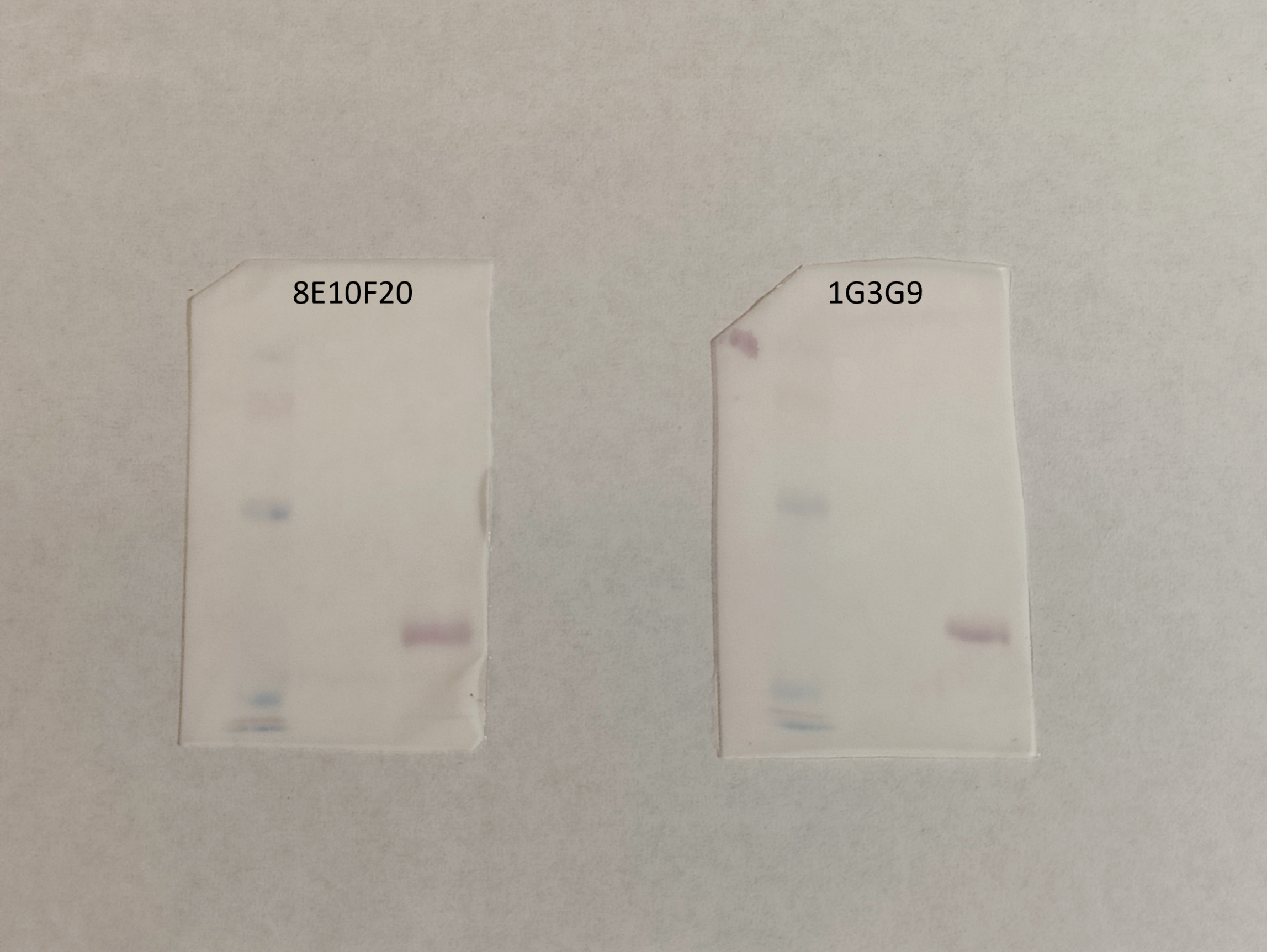


**Supplementary Figure S8.** Raw image for Figure 6A. Western blot of PVDF electrotransferred rVlsE vs. mAbs.
